# Supplementary material for: Polariton lattices as binarized neuromorphic networks
Source: Light Sci Appl. 2025 Jan 16;14:52. doi: 10.1038/s41377-024-01719-4 (PMC11739516; doi:10.1038/s41377-024-01719-4)
Supplement: Supplementary file 1 — Supplementary Information for Polariton lattices as binarized neuromorphic networks [file 41377_2024_1719_MOESM1_ESM.pdf]

Supplementary Information for
Polariton lattices as binarized neuromorphic networks

Evgeny Sedov[1,2,3,4]*, Alexey Kavokin[1,3,4]

[1]Spin-Optics laboratory, St. Petersburg State University, St. Petersburg 198504, Russia

[2]Stoletov Vladimir State University, Vladimir 600000, Russia

[3]School of Science, Westlake University, 600 Dunyu Road, Hangzhou 310030, Zhejiang Province, China

[4]Abrikosov Center for Theoretical Physics, Moscow Institute of Physics and Technology, Dolgoprudnyi, Moscow Region 141701 Russia

S1 Structural nonlinearity in networks with linear neurons

S1.1 Structural nonlinearity from random pairing in the dyad lattice

The random pairing of adjacent condensates into dyads introduces a form of structural nonlinearity. Although each dyad performs a linear operation (an OR operation in this case), the overall effect of combining multiple such operations leads to a nonlinear transformation of the input space. This is akin to feature engineering, where the new features (outputs of OR gates) enable a linear classifier to achieve nonlinear decision boundaries.

In the context of the dyad lattice, while each dyad neuron performs a linear operation, the random arrangement and the combination of these operations across the network introduce nonlinearity. This setup allows the network to capture and model complex patterns, similar to how hidden layers in neural networks use nonlinear activation functions to achieve nonlinear separability.

Each neuron in the hidden layer functions as an OR gate, inherently providing a binary output based on its two randomly chosen binary inputs. Despite individual neuron outputs being dictated by a simple linear decision boundary (in the context of binary operations), the arrangement and aggregation of multiple such neurons introduce complexity into the system as a whole. Each neuron can be visualized as sampling and encoding different parts of the input space. Because the input pairs are chosen randomly and can overlap among neurons, the collective output pattern of the hidden layer, which comprises multiple such OR operations, creates a new representation of the input space.

When these varied outputs from numerous neurons enter the linear classifier, they represent a transformation of the input space into a new feature space where the axes are the outputs of the OR gates. The linear classifier then operates in this transformed space. The key here is that while the classifier itself applies a linear function to the outputs from the hidden layer, the space it operates in has been transformed by the combined OR operations, which effectively re-encode the inputs in a way that can make linearly inseparable data (like XOR) separable in this new feature space.

Thus, the nonlinearity of the network doesn't come from individual OR neurons, which are linear, but from how the inputs are pre-processed and encoded by the collective arrangement and interaction of these neurons. Each neuron effectively creates a "micro-boundary" in the input space, and the ensemble of such boundaries, which vary randomly in location due to the random pairing of inputs, configures a complex, linearly inseparable dataset into a form that the final linear classifier can process. This results in a network capable of handling nonlinear separations at the input level, even though the final decision-making (classification) step is linear.

*Email: evgeny_sedov@mail.ru

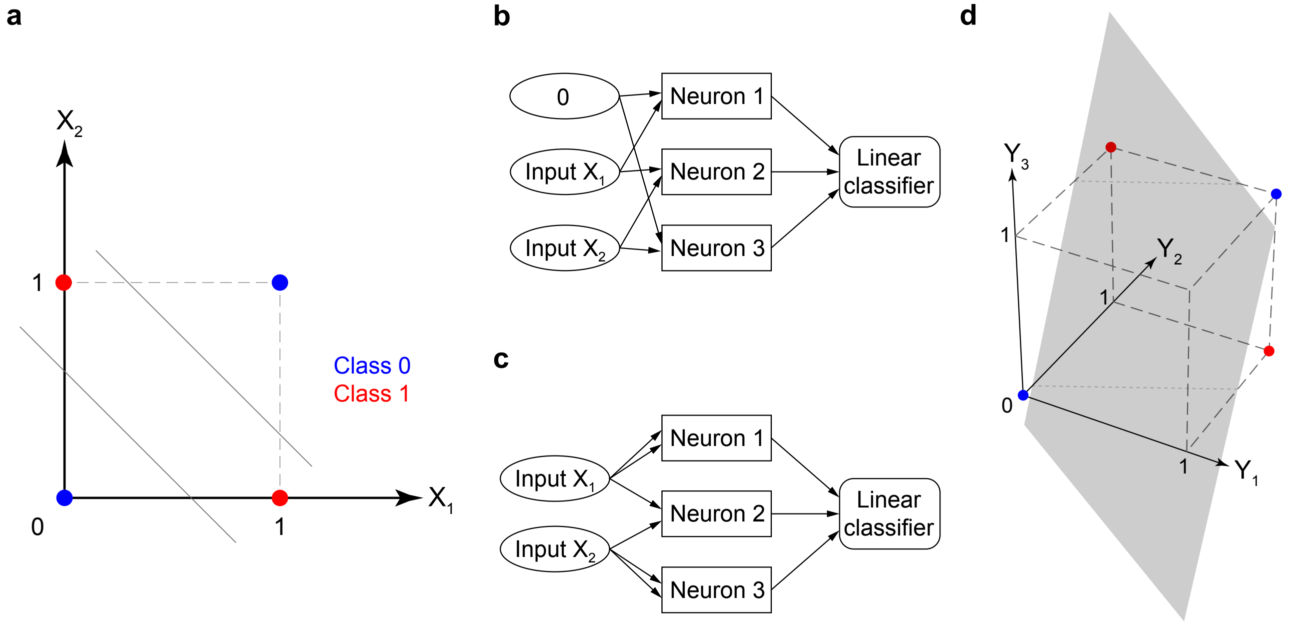

Figure S1: **Overcoming the linear inseparability of the XOR operation through the introduction of a hidden layer composed of linear OR-gate neurons. a** Illustration of the linear inseparability of the XOR operation. The two classes of outputs, 0 (blue) and 1 (red), cannot be separated by a single straight line in the input space. **b**,**c** Schematics of two alternative simple neural networks with hidden layers composed of three neurons functioning as OR gates, considered for solving the XOR problem. **d** Linear separation of the two classes, 0 and 1, in the feature space of outputs from the neurons in the hidden layer

S1.2 Demonstrating nonlinearity: Solving the XOR problem with linear neurons

The emergence of nonlinearity due to the structure of the hidden layer can be illustrated through a simple example of solving the so-called XOR problem. The XOR (exclusive OR) problem involves two binary inputs and one binary output. The output is true if exactly one of the inputs is true. The input-output pairs are as follows:

- $(0,0) \to 0$,
- $(0,1) \to 1$,
- $(1,0) \to 1$,
- $(1,1) \to 0$.

As one can see, the outputs of the XOR operator reduce to two classes: 0 and 1. The XOR problem is well-known for being linearly inseparable, meaning no single linear decision boundary can correctly classify all possible input-output pairs, see Fig. S1**a**. However, by using a network of OR gates to transform the input space, followed by a linear classifier, we can solve the XOR problem, confirming the introduced nonlinearity through the network structure.

For this purpose, we use a neural network with two inputs, X_1 and X_2, constructed according to the principles outlined in the main text, consisting of just three neurons, see schematic in Fig. S1**b**. The setup of the hidden layer is as follows.

- **Neuron 1** OR(X_1, 0): One input is signal X_1, and the other input is a null signal (0).
- **Neuron 2** OR(X_1,X_2): One input is signal X_1, and the other input is signal X_2.
- **Neuron 3** OR(X_2, 0): One input is signal X_2, and the other input is a null signal (0).

In the architecture of the network illustrated in Fig. 1**a** and 1**b** of the main text, neurons 1 and 3 correspond to those at the edge of the lattice. Alternatively, these neurons can be replaced with neurons OR(X_1,X_1) and

OR(X_2,X_2), which have the same signal at both inputs, see Fig. S1**c**. This is permissible in our proposed architecture because the signals to the inputs of the neurons are assigned randomly.

The hidden layer transforms the original input space into a higher-dimensional feature space. In doing so, the vector of inputs $\mathbf{X} = (\mathrm{X}_1, \mathrm{X}_2)$ is projected into the feature space, $\mathbf{X} \to \mathbf{Y}$, where $\mathbf{Y} = (\mathrm{Y}_1, \mathrm{Y}_2, \mathrm{Y}_3)$ is the vector of outputs of Neurons 1, 2, and 3, as follows:

- $(0,0) \to (0,0,0)$,
- $(0,1) \to (0,1,1)$,
- $(1,0) \to (1,1,0)$,
- $(1,1) \to (1,1,1)$.

Dimensionality of the feature space corresponds to the number of neurons in the hidden layer. Fortunately, the use of only three neurons in our example allows us to visualize the signals in the feature space, see Fig. S1**d**. The figure allows to intuitively confirm that in the feature space, the two classes of outputs can be effectively separated by a plane. This indicates that the separation can be successfully achieved by a linear classifier.

Let's support our intuition with simple calculations. Let's denote the classes to be separated:

- **Class 0**: (0,0,0) and (1,1,1).
- **Class 1**: (1,1,0) and (0,1,1).

The separating plane can generally be described by the following equation:

$$f(\mathrm{Y}_1, \mathrm{Y}_2, \mathrm{Y}_3) = w_1\mathrm{Y}_1 + w_2\mathrm{Y}_2 + w_3\mathrm{Y}_3 + b$$

with $w_{1,2,3}$ and b being weights and a bias, respectively. For the linear classification, we need a function, which

- for Class 0: $f(0,0,0) \geq 0$ and $f(1,1,1) \geq 0$,
- for Class 1: $f(1,1,0) < 0$ and $f(0,1,1) < 0$.

With the weights $w_{1,3} = 1$, $w_2 = -2$, and a bias $b = 0.5$, the equation becomes

$$\mathrm{Y}_1 - 2\mathrm{Y}_2 + \mathrm{Y}_3 + 0.5 = 0.$$

For these weights, the function produces the following outputs for each input combination:

- $f(0,0,0) = 0.5$,
- $f(1,1,1) = 0.5$,
- $f(1,1,0) = -0.5$,
- $f(0,1,1) = -0.5$.

These estimations confirm that the linear classifier can effectively separate the two classes using this plane.

S1.3 The contribution of randomization to differentiating states in feature space

As demonstrated above, nonlinear transformation of the input space can be achieved using linear neurons through a specific arrangement of their interactions with input signals. In our study, the mechanism of randomization plays a pivotal role in configuring connections within our neural network. As the dimensionality of the hidden layer increases — marked by a higher number of neurons — the potential for diverse randomization scenarios also grows. This randomization, however, does not guarantee the desired arrangement. Nevertheless, it is possible to assess the extent to which it facilitates nonlinearity.

Let us again treat the second-order XOR problem, which involves two binary input signals that are categorized into two distinct classes based on the XOR operation. The pair of signals is transformed to a $n_{\mathrm{in}} \times n_{\mathrm{in}}$ square input lattice and processed through a hidden layer of randomly arranged $N_{\mathrm{d}} \leq n_{\mathrm{in}}^2 + 2n_{\mathrm{in}} + 1$ neurons, as detailed in the main text. The focus of our analysis lies in examining the corresponding states, binary vectors of length N_{d}, in the feature space. We examine these vectors to determine if any vectors from different classes overlap. The presence of identical vectors across classes would clearly indicate that the classes cannot be linearly separated.

It's crucial to highlight that the absence of overlapping vectors does not conclusively prove class separability. However, it does demonstrate the efficacy of the nonlinear transformation in creating unique state sets for each class, thereby facilitating a deeper analysis of the system's inherent nonlinearity. To rigorously assess the impact of randomness, we perform repeated simulations (1000 runs each) across lattices of varying dimensions n_{in}.

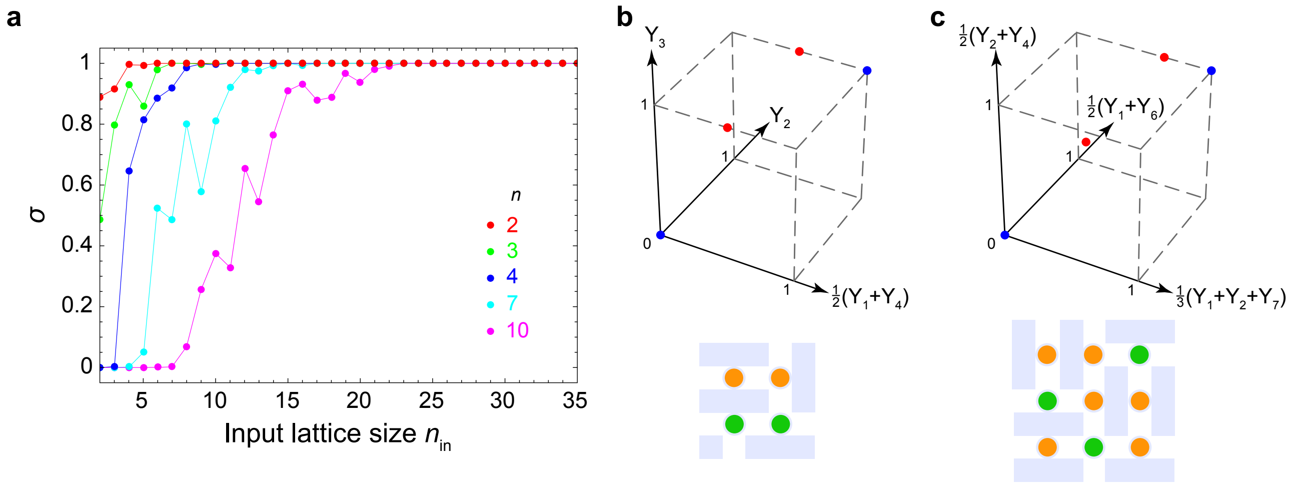

Figure S2: **Emergence of unique, class-specific states in feature spaces for the generalized XOR problem.** **a** The dependence of the fraction of successful runs, σ, that led to the emergence of unique sets of states for each class in the feature space for the n-th-order XOR problem, on the lattice size n_{in}. **b**,**c** Examples of non-overlapping states in feature spaces of dimensions 4 (**b**) and 9 (**c**), respectively, projected into three-dimensional space, with states corresponding to class 0 (blue) and class 1 (red) of the second-order XOR problem. The feature space reflects the configurations of neuron lattices schematically shown in the lower panels of the corresponding figures. Blue rectangles depict neurons, similarly to Fig. 1 in the main text. Orange and green circles indicate signals arranged according to the input signal lattices of size $n_{\text{in}} = 2$ (**b**) and $n_{\text{in}} = 3$ (**c**)

Figure S2**a** illustrates with a red line the dependence of the fraction of successful runs that led to the creation of unique sets of states for each class in the feature space, on the lattice size. It can be clearly seen that, first, even for a lattice of the smallest possible size (2×2), containing 4 neurons, the probability of a successful run with random connections is close to 0.9. Second, as the lattice size increases, this probability approaches 1 for a 6×6 lattice within the studied data sample. Figures S2**b** and S2**c** illustrate successful runs, depicting non-overlapping states in feature spaces of dimensions 4 and 9, respectively, projected into three-dimensional space.

The XOR problem can be generalized to higher dimensions. Let n be the number of input variables, where each input X_i can take a binary value of either 0 or 1, with i ranging from 1 to n. By increasing the number of inputs, we can proportionally increase the number of classes. For the generalized XOR problem, the classes are defined as follows:

1. Compute the sum of the input values: $S = \sum_{i=1}^{n} \mathrm{X}_i$.

2. Apply the MOD operation to the sum to categorize the inputs into n different classes: Class $= S \mod n$.

It can be verified that such a scheme for class determination is valid for the second-order XOR problem discussed above.

Figure S2**a** shows the dependence of the fraction of successful runs on the lattice size for XOR problems of different orders n. A common characteristic of all the dependencies is that the probability of achieving unique sets of states for each class in the feature space rapidly approaches unity as the lattice size increases and the impact of randomization on the connections strengthens. Notably, this probability starts deviating from zero much earlier for significantly smaller lattice sizes. This allows for the identification of a successful configuration of the hidden layer and the randomization mask for input signals, which can be used in further studies. We want to emphasize once again that this characteristic does not necessarily indicate linear separability of the states in the feature space. However, it signifies the creation of unique sets of states for each class, achieved due to the nonlinearity introduced by the specific structure and connectivity of the network, which transforms the input signals in a way that facilitates this separation.

S1.4 Similarity of states

Universal neural networks designed to solve a wide range of tasks are certainly advantageous, but this versatility comes at the cost of increased architectural complexity. In contrast, specialized neural networks tailored for specific tasks can be much simpler and more efficient in their design. The complexity of a neural network's architecture is directly related to the complexity of the task it aims to solve. This complexity can be objectively assessed using various criteria.

One such criterion is the similarity of states within a single class. If the states within a class are highly similar, their common features will be more pronounced. Conversely, if the similarity is low, the distinguishing features will be less prominent, requiring more diffuse features to achieve effective separation.

In previous sections, we have discussed the performance of the network in solving the higher-order XOR problem. However, we want to emphasize that this problem is significantly more challenging than the tasks of handwritten digit recognition or spoken words recognition, considered in the main text. Therefore, the positive conclusions about the network's performance can be applied with even greater confidence and certainty to the digit recognition task, dispelling any doubts about its efficacy.

The similarity of states represented as binary vectors can be evaluated using a metric known as the normalized Hamming distance. This metric provides a quantitative measure of how similar or dissimilar two binary vectors are. The Hamming distance between two binary vectors is the number of positions at which the corresponding bits differ. The Hamming distance between two vectors of length n, $\mathbf{v} = (v_1, v_2, ..., v_n)$ and $\mathbf{w} = (w_1, w_2, ..., w_n)$, is calculated as

$$l_{\mathrm{H}}(\mathbf{v}, \mathbf{w}) = \sum_{i=1}^{n} |v_i - w_i|. \tag{S1}$$

We can introduce the normalized Hamming distance

$$L_{\mathrm{H}}(\mathbf{v}, \mathbf{w}) = \frac{l_{\mathrm{H}}(\mathbf{v}, \mathbf{w})}{n}, \tag{S2}$$

which quantifies the ratio of differing positions between two binary vectors relative to their length. This metric can range from 0 to 1:

- A value of 0 indicates that the vectors are identical, meaning there is complete similarity.
- A value of 1 indicates that the vectors are completely different, meaning there is no similarity.

Intermediate values indicate varying degrees of similarity. The normalized Hamming distance allows one to compare the similarity of classified states across different tasks, even when the lengths of the states differ between tasks.

For XOR problems of different orders n, we find the average Hamming distance $\langle L_{\mathrm{H}}^{(j)} \rangle$ for each class j. Figure S3**a** illustrates the Hamming distance $\langle\langle L_{\mathrm{H}} \rangle\rangle = \frac{1}{n}\sum_{j=0}^{n-1} \langle L_{\mathrm{H}}^{(j)} \rangle$, averaged across all classes, plotted against the order n of the XOR problem. The shaded area indicates the range of $\langle L_{\mathrm{H}}^{(j)} \rangle$ values for different classes. The upper boundary, $\langle L_{\mathrm{H}} \rangle_{\max} = \langle L_{\mathrm{H}}^{(0)} \rangle$, corresponds to Class 0, which always includes only two states that are maximally different from each other: one consisting entirely of zeros and the other entirely of ones. On average, the similarity of states within a class increases (Hamming distance decreases) with the increase in the order of the problem n, approaching an asymptote just below 0.4.

Figure S3**b** illustrates the similarity of states within a class for the task of handwritten digit recognition using the MNIST dataset. We vary the size of the images and evaluate the Hamming distance. The dependence of $\langle\langle L_{\mathrm{H}} \rangle\rangle$ on the image size is shown by the black curve in the figure. Variation of $\langle L_{\mathrm{H}} \rangle$ across the layers is shown by the gray shade. As can be seen, the average Hamming distance changes slowly with varying image sizes and does not exceed 0.18. This value is at least twice as small as that for high-order XOR problems. In addition, in Fig. S3**b**, the maximum average distance does not exceed 0.25. All this indicates significantly greater similarity of states within a single class in the digit recognition problem and a more pronounced distinctiveness of their features, which facilitates their separability. It is noteworthy that for the Hamming distance, there is a local minimum in the dependence when the image size is close to the original (28×28).

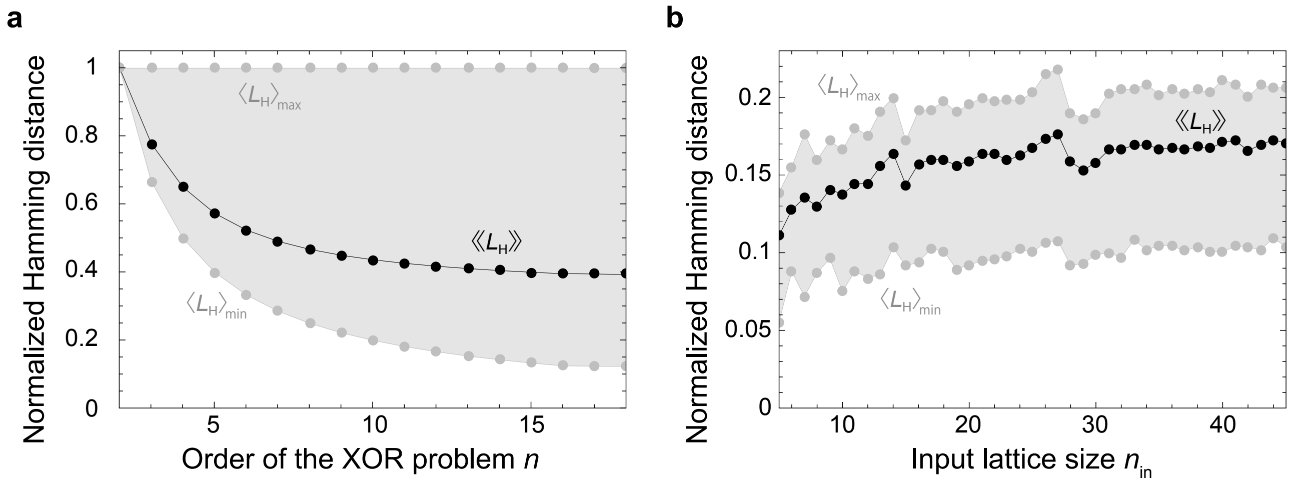

Figure S3: **Scaling of Hamming distances for the XOR problem and the handwritten digit recognition task.** **a** The average Hamming distance, $\langle\langle L_{\mathrm{D}}\rangle\rangle$, between states within a single class for XOR problems, plotted against the order n of the problem (black). The gray shadow indicates the variation of the average Hamming distance, $\langle L_{\mathrm{D}}^{(j)}\rangle$, across the classes. **b** The average Hamming distance within a single class for the task of handwritten digit recognition, plotted against the image size

S2 Handwritten digits classification task without the polariton lattice layer

In the main text of the paper, when evaluating the accuracy of handwritten digit classification, we benchmarked the efficacy of our proposed approach against the baseline accuracy achieved by software-based classification of original MNIST dataset images. This included both grayscale and binarized versions of the images, where the reference standard was the accuracy obtained using logistic regression without regularization on the original image size of 28×28 pixels. However, unlike the benchmark where the input image size is fixed, our study treats image size as a variable.

To substantiate the fairness of our results comparison with the benchmark, we conducted a series of accuracy evaluations based on different image sizes. The findings are presented in Fig. S4. Specifically, we assessed how the classification accuracy for grayscale images (blue) and binarized images (green) varied under conditions without (Fig. S4**a**) and with (Fig. S4**b**) randomization. In the absence of randomization, image resizing was conducted through a straightforward interpolation method, which entailed inserting intermediate points to adjust the image scale. Conversely, when randomization was present, image enlargement followed the procedure described in the main text: randomly selecting elements from the original image and integrating them into the input signal array. In all cases, classification was carried out using logistic regression without regularization, implemented via Wolfram Mathematica version 14.0.

In both scenarios — the classification of grayscale images and binarized images — the accuracy of grayscale classification consistently surpasses that of the binarized images, albeit the difference seldom exceeds two percent and is often within one percent. Furthermore, the relationship between accuracy and lattice size exhibits a saturation behavior across all scenarios. Specifically, while accuracy varies with smaller lattice sizes, it tends to stabilize and shows minimal change as the lattice size increases. Lastly, at no point does the accuracy achieved with any lattice size approach the levels obtained with our system employing a polariton lattice.

The result obtained can be attributed to several factors. Firstly, the original MNIST images are relatively low resolution at 28×28 pixels. When these images are resized to larger dimensions, no new information is added; rather, the images are simply interpolated or randomized to fill the larger size. This process does not enhance the inherent features of the digits, which are critical for accurate classification. Instead, it can introduce noise or redundant data, which does not contribute to, and may even detract from, the classification performance.

Moreover, logistic regression, being a linear classifier, has inherent limitations in capturing complex patterns in the data, especially when the input size increases without adding meaningful information. As the lattice size grows, the model may face an overfitting issue where it learns the noise instead of the actual signal, leading

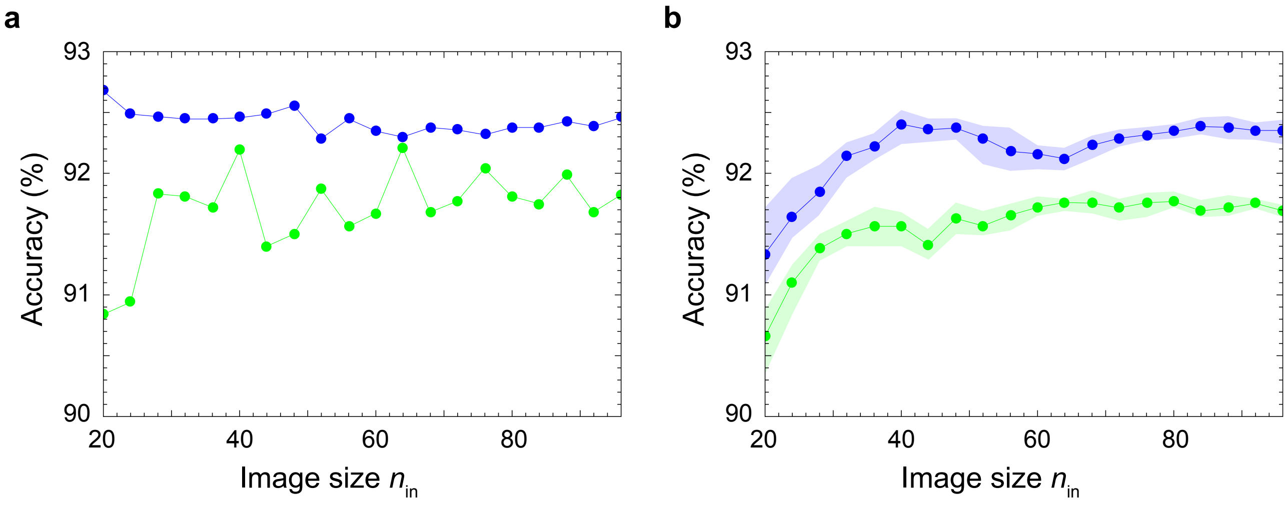

Figure S4: **Influence of image resizing and binarization on MNIST classification accuracy without the polariton layer.** The changes in classification accuracy for grayscale (blue) and binarized (green) images from the MNIST dataset as their sizes are varied. Image resizing was performed through **a** a straightforward interpolation method, involving the insertion of intermediate points to adjust the image scale, and **b** combined with randomization, following the methodology employed in the main text

to a plateau in accuracy improvements. This saturation effect underscores the necessity of incorporating more sophisticated techniques or additional layers, such as the polariton lattice layer in our proposed system, to effectively harness the potential of larger input sizes for improved classification performance.

In the main text of the paper, logistic regression is used to emphasize the nonlinearity introduced by the polariton lattice. This approach highlights the significant advantage of our system, which effectively utilizes the polariton lattice to achieve superior classification results, demonstrating how it can enhance the capabilities beyond what a linear classifier alone can achieve.

S3 The influence of signal pump pulse duration on the operation of artificial polariton neurons

The polariton condensates that form the hidden layer lattice of the neural network spontaneously acquire mutual coherence through the exchange of ballistically propagating quasiparticles, resulting in the formation of interference patterns. The interference pattern within each isolated polariton dyad, particularly its parity, is fundamental to the operation of artificial polariton neurons. In the absence of signal pulses, the parity of the interference pattern in the dyad is primarily determined by the width and duration of the optical pump pulses, as well as the distance between the condensates in the dyad. For the given parameters of the system, our numerical experiments show that the required parity of the interference patterns in the dyads, combined with the clarity and illustrative nature of subsequent calculations, can be achieved with a lattice period $d = 12\,\mu$m, pump pulse width of $w_1 = 2.2\,\mu$m, and pulse duration of $w_{\tau 1} = 5\,$ps. Without sacrificing generality, we select this combination of parameters as the reference for our calculations and analyses in the paper.

The parity change of the interference patterns is achieved through signal pulses. The parameters of these pulses are chosen to ensure they have sufficient influence on the interference pattern to alter its parity, and that this change can be successfully detected. In the main text, we selected signal pulses with the same width as the lattice condensate pump pulses. In our calculations, we also assume that the delay between the pulses is zero, meaning the peaks of the signal pulses and the pump pulses coincide in time. For signal pulse duration $w_{\tau 2} = 8$ ps, we obtained an optimal intensity ratio that maximizes the distinguishability of the ON and OFF states characterized by the parameter $\Delta\tilde{I}$, see blue line in Fig. 2 in the main text. The parameter is determined as a difference between the minimum intensity of output signals from dyads expected to be in the ON state, $\mathrm{Min}(I_{\mathrm{ON}})$, and the maximum intensity of signals from dyads expected to be in the OFF state, $\mathrm{Max}(I_{\mathrm{OFF}})$, normalized by the latter, $\Delta\tilde{I} = [\mathrm{Min}(I_{\mathrm{ON}}) - \mathrm{Max}(I_{\mathrm{OFF}})]/\mathrm{Max}(I_{\mathrm{OFF}})$.

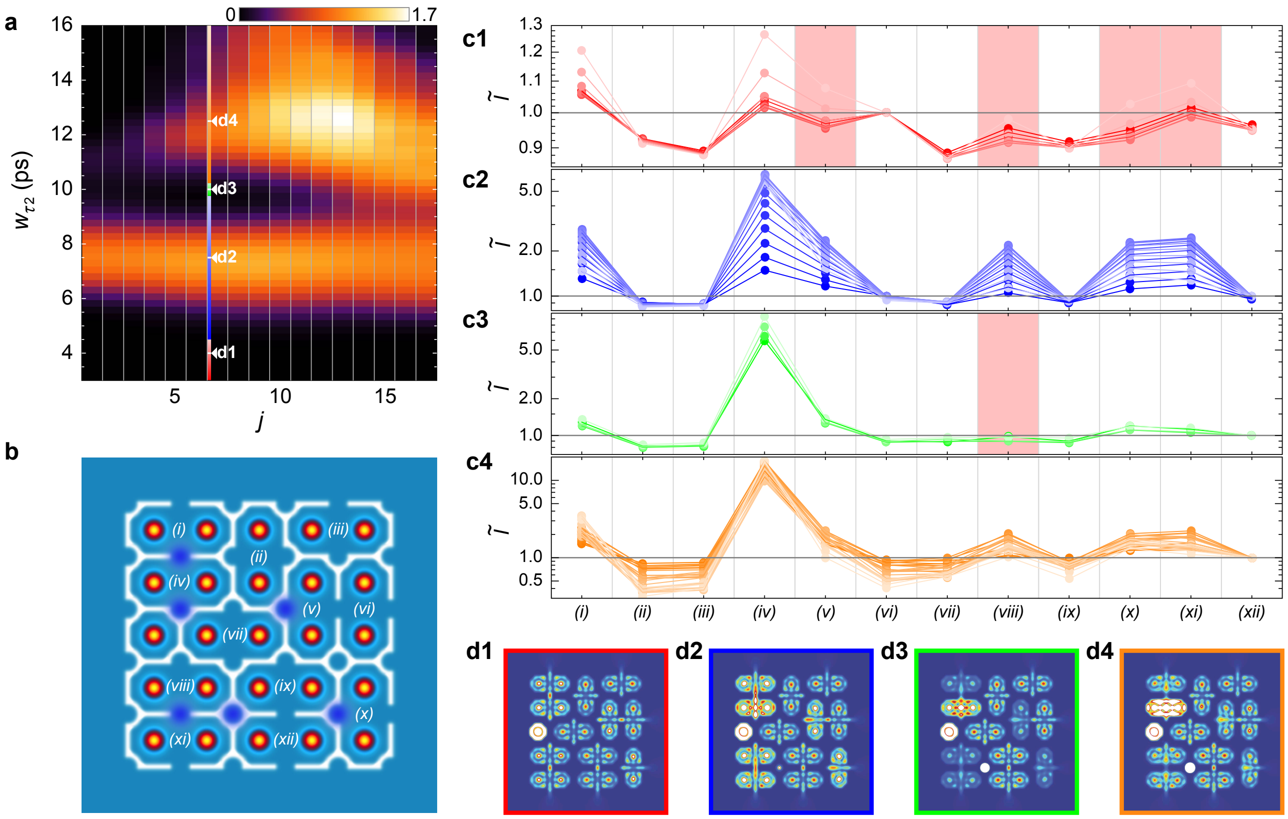

Figure S5: **Controlling the distinguishability of neuron output signals through variations in pump pulse durations and intensities. a** The dependence of distinguishability, $\Delta\tilde{I}$, of the OFF and ON signals on the duration of the signal pump pulses, $w_{\tau 2}$, at certain pump intensities (P_{10}, P_{20}) corresponding to points $j = 1, 2, ...17$ in Fig. 2**d** in the main text. The duration of the condensate pump pulses $w_{\tau 1}$ is taken 5 ps. Values of $\Delta\tilde{I} \leq 0$ are colored in black. The colored stripes at $j = 7$ indicate the ranges of variation of $w_{\tau 2}$ in **c**1–**c**4. The white markers indicate the values of the parameters used for **d**1–**d**4. **b** The profiles of the non-resonant pump spots (main color scheme), the trapping potential profile for isolating dyads (white) and the control beam profiles (blue) for toggling dyads between ON and OFF states. The indices *(i)* to *(xii)* enumerate artificial neurons in the polariton lattice. **c**1–**c**4 Variation of the reduced intensity of output signals, $\tilde{I}$, in each neuron from *(i)* to *(xii)* at the pump intensities (P_{10}, P_{20}) corresponding to $j = 7$, for different durations of the signal pump pulses $w_{\tau 2}$, indicated by color stripes in **a**. Lines connect points obtained in a single numerical experiment for a given value of $w_{\tau 2}$. The intensities are normalized by the maximum intensity of signals from dyads expected to be in the OFF state. The reduced intensity of the corresponding dyad is equal to 1 (indicated by gray lines). Dyads whose states do not match the expected states under the given excitation conditions are highlighted with a red background. **d**1–**d**4 Time-integrated spatial distribution of the polariton density at different durations of the signal pump pulses $w_{\tau 2}$ indicated in **a**

In this supplemental section, we investigate how the duration of signal pulses affects the distinguishability of ON and OFF signals. The color map in Fig. S5**a** shows how at the intensities of the pulses (P_{10}, P_{20}), corresponding to points j ranging from 1 to 17 in Fig. 2 of the main article, the distinguishability $\Delta\tilde{I}$ varies as a function of the signal pulse duration $w_{\tau 2}$. It is evident that the dependence of $\Delta\tilde{I}$ on $w_{\tau 2}$ is non-monotonic. Several characteristic zones are identified. To illustrate these zones, Figs. S5**c**1–S5**c**4 show the changes in the output signal intensities of each neuron from *(i)* to *(xii)* as a function of $w_{\tau 2}$ in each of these zones. The intensities are normalized by $\mathrm{Max}(I_{\mathrm{OFF}})$.

For $w_{\tau 2}$ less than $w_{\tau 1}$, the ON and OFF states are indistinguishable. According to Fig. S5**c**1, most neurons (highlighted in red), which are excited by a single signal pulse, incorrectly remain in the OFF state instead of switching to the ON state. Clearly, the energy of the short pulse is insufficient to switch the states. The exception is neuron *(i)*, which correctly switches to the ON state. This is likely due to the contribution of the input signal located on the same line but two neurons away, namely near *(iv)*. This indicates the presence of

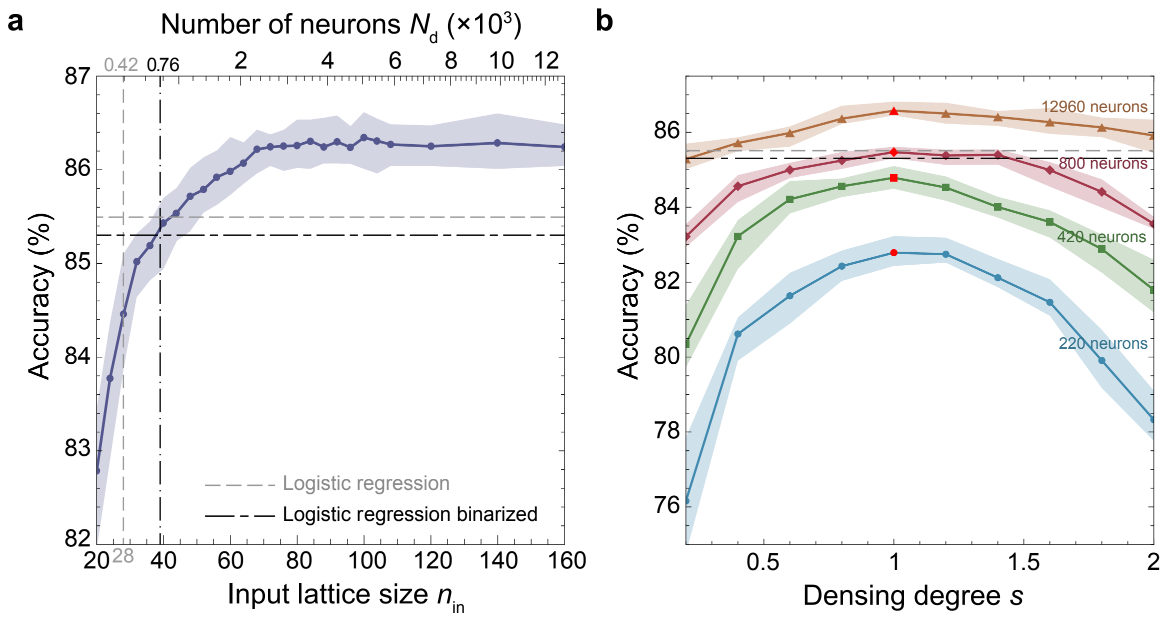

Figure S6: **Evaluation of the Fashion-MNIST image recognition by the polariton neuromorphic network. a** The recognition accuracy in dependence on the size of the input signal lattice $n_{\rm in}$ (lower scale) or the number of neurons (dyads) in the hidden layer $N_{\rm d}$ (upper scale). **b** The recognition accuracy in dependence of the overlap degree s of the input signal for square polariton lattice systems of different size $n_{\rm in}$ with different numbers of neurons in the interaction layer: $n_{\rm in} = 20$ with 220 neurons (blue), $n_{\rm in} = 28$ with 420 neurons (green), $n_{\rm in} = 39$ with 800 neurons (red), and $n_{\rm in} = 160$ with 12960 neurons (brown). All designations in the panels are the same as in Fig. 4 in the main text

crosstalk between neighboring dyads, which in these conditions is strong enough compared to other effects. The characteristic distribution of polariton density in this pumping regime is illustrated in Fig. S5**d**1.

The next zone, which for our parameters lies in the range from about 5 ps to about 9 ps, corresponds to the primary operating mode of the polariton neurons, see also Fig. S5**c**2. In this zone, all states are distinguishable, with $\Delta\tilde{I}$ varying within the specified pulse duration and exhibiting a pronounced maximum. As observed, crosstalk does not manifest itself and does not cause false state switches.

As the duration of the signal pulses continues to increase, the ON and OFF states again become indistinguishable. As shown in Figs. S5**c**3 and S5**d**3, this is primarily due to neuron *(viii)*. One input of this neuron receives the control signal, while the second input remains unexcited and is isolated from other neurons by a barrier (unlike, e. g., neuron *(i)*, which has both inputs open). The inability of neuron *(viii)* to switch states may be attributed to negative self-interference of the signal pulse under conditions of reflection from the barrier at the second input. Subsequent increases in $w_{\tau 2}$ once again shift the system into a regime with distinguishable ON and OFF states, see Figs. S5**c**4 and S5**d**4.

Thus, the operation of the artificial neurons is fundamentally based on the interference effect of polariton condensates within each dyad. However, this same interference effect is also crucial in the interaction of neurons with signal pulses, barriers, and each other (neuron crosstalk). Therefore, when selecting the parameters of the pump pulses, it is important to ensure that unwanted crosstalk and similar effects are either suppressed or rendered negligible against the beneficial interactions between neurons and control signals. Fortunately, as demonstrated by our numerical experiments, it is feasible to choose parameters that meet these requirements and ensure their fulfillment over a wide range of pump intensities and pulse durations. Furthermore, the crosstalk effects, which are currently considered parasitic, could form the basis of future research in this field. These effects can be transformed from undesirable interactions into useful mechanisms that facilitate communication between more distantly located neurons, potentially enhancing the capabilities of the neural network.

S4 Fashion-MNIST dataset analysis

We applied the proposed network to the Fashion-MNIST dataset. The dataset features 70000 images across 10 categories of fashion items, including footwear, bags and clothing types, maintaining the same structure as the MNIST dataset with 60000 training images and 10000 testing images. Fashion-MNIST was developed as a more challenging and diverse alternative to the traditional MNIST, providing images that represent real-world scenarios rather than simple digit recognition. This complexity is crucial for testing the robustness of our neuromorphic network, as it demands higher pattern recognition capabilities and differentiation between more intricate and variable image features.

Building on our examination of the Fashion-MNIST dataset, our findings are summarized in Fig. S6. As anticipated, the accuracy of image classification increases monotonically with the number of neurons involved, achieving a notable accuracy of 86.5% at its maximum. The saturation of the dependency, shown in Fig. S6**a**, mirrors our observations with the standard MNIST dataset. Our polariton network surpasses performance of conventional linear classifiers, which confirms the network's effective functioning through increased structural nonlinearity as more neurons are involved. However, it is notable that while the performance enhancement is remarkable, the overall gain in accuracy is less dramatic than observed with the simpler MNIST dataset. Additionally, the saturation in accuracy gains occurres at a smaller lattice size, approximately $n_{\text{in}} = 70$ as opposed to around 100 for MNIST. These characteristics underscore the inherent complexity and variability of the Fashion-MNIST dataset.

An notable observation from our study pertains to the application of input signal densing. As depicted in Fig. S6**b**, unlike with the MNIST dataset, adjusting the densing degree s for the Fashion-MNIST does not yield improvements in classification accuracy, regardless of the neuron count. This observation, along with the earlier discussed saturation of the accuracy curve, can be attributed to the fact that the average filling of the initial binarized matrix in Fashion-MNIST is estimated at approximately 0.5, compared to 0.19 for MNIST. This implies a natural balance between '0' and '1' signals in the original images, a balance that the densing technique aims to achieve.

S5 Understanding Mel-Frequency Cepstral Coefficients

The Mel scale is a perceptual scale of pitches judged by listeners to be equal in distance from one another. This scale is crucial for processing audio because it closely approximates the human auditory system's response to different frequencies. It emphasizes lower frequencies which are more critical for understanding spoken language, as these frequencies carry more phonetic information than higher frequencies. By mapping real frequency values to the Mel scale, speech recognition systems can focus on the most phonetically significant parts of an audio signal. Transformations of a sound file consist in converting it into a matrix of Mel Frequency Cepstral Coefficients (MFCCs) [S1, S2]. The process of deriving MFCCs involves the following steps:

- *Digital Conversion*: The audio file is first converted into a digital signal array, which represents the sound wave in a format that can be processed computationally. See an example of the raw wave plot of the word "seven" in Fig. S7**a**
- *Frame the Signal*: The sound is divided into short frames, each about 40 ms long.
- *Apply the Fourier Transform*: A Fourier transform, often the Fast Fourier Transform (FFT), is applied to each frame to transform the time-domain signal into the frequency domain.
- *Mel Filter Bank Processing*: The power spectrum from the FFT is passed through a Mel filter bank. The filters in this bank are spaced linearly at low frequencies and logarithmically at high frequencies, reflecting the human ear's varying sensitivity across its frequency range.
- *Logarithmic Scaling*: The logarithm of the energy output from each filter is calculated to simulate the logarithmic perception of loudness and pitch in human hearing.
- *Discrete Cosine Transform (DCT)*: The log-transformed filter bank energies undergo DCT. This step decorrelates the energy values and compresses them into the final set of coefficients, the MFCCs. See visualization of the MFCC feature matrix in Fig. S7**b**.
- *Binarization*: After calculating the MFCCs, the final preprocessing step involves binarizing the coefficients to simplify further processing, especially for networks designed to work with binary data. This is achieved

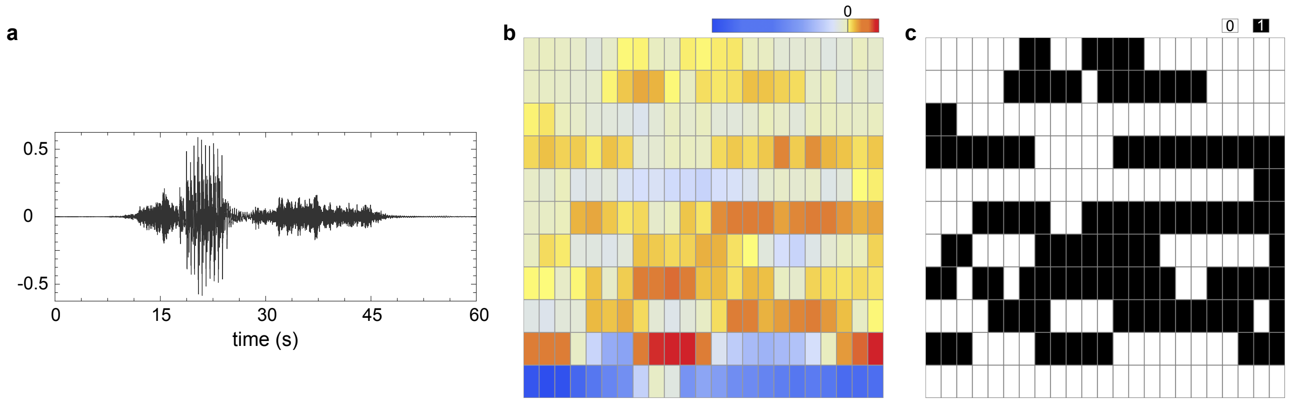

Figure S7: **Illustration of data processing applied to spoken word samples from the Speech Commands dataset.** **a** An example of the raw wave plot, **b** the MFCC feature matrix, and **c** the binarized matrix of the word "seven" from the Speech Commands dataset [S3, S4]

by applying a simple rule: coefficients greater than zero are set to 1, and those less than or equal to zero are set to 0.

Assessments indicate that, on average, the filling of the binarized feature matrix is approximately 0.51. This implies that the number of positive and negative elements in the MFCC matrix are roughly equal on average. This can be attributed to the nature of the cosine transform utilized in MFCC calculations, which typically produces a symmetric distribution of coefficients. The MFCC computation involves DCT of the logarithmic Mel-spectrogram. This logarithmic transformation normalizes amplitude variations in the sound signal, and the subsequent DCT captures the frequency content of the signal. Given that the logarithmic scale can yield both positive and negative values depending on the energy distribution across the Mel-scale filters, and considering that DCT inherently includes a mixture of cosine functions oscillating around zero, this process generally results in a balanced distribution of MFCC values around zero.

S6 Data Binarization and Signal Densing

In implementing our network architecture, we address the critical step of binarizing input data, which is particularly relevant when working with datasets like MNIST. Each image in the MNIST dataset comprises an array of pixel intensities, where each pixel value is a real number ranging from 0 (black) to 1 (white). To adapt these images for processing in our binary neuromorphic network, we convert them into binary images through a thresholding operation.

The threshold value, denoted as b, can be chosen arbitrarily within the interval $[0, 1)$. During binarization, pixels with intensities less than or equal to b are assigned a value of 0, while pixels with intensities greater than b are assigned a value of 1. The choice of threshold b directly influences the degree of activation within the input array — that is, the proportion of pixels assigned a value of 1 versus those assigned 0.

A lower threshold results in fewer activated pixels (more 0's), while a higher threshold increases the number of activated pixels (more 1's). However, this relationship holds true only up to a certain limit, as the dependence between the threshold value and the degree of activation is not uniform and depends on the structural characteristics of the data being processed. Specifically, in the case of the MNIST dataset, the dependence of the average filling degree, that is, the ratio of the number of 1's to the total number of pixels, on the threshold b is illustrated in Fig. S8**a**.

In the MNIST dataset, the majority of each image consists of pixels with zero intensities, representing the background, while the handwritten digits occupy a smaller portion with non-zero intensities. As a result, even at a threshold of $b = 0$, where only pixels with zero intensity are assigned a value of 0, the filling degree does not exceed approximately 0.19. As we increase the threshold b, the filling degree decreases even further, since more pixels with low intensities (previously assigned a value of 1) are now classified as 0. Examples of binarized images at different thresholds before and after randomization are shown in Figs. S8**b**1 and S8**b**2.

To enhance neuron activation and strengthen nonlinearity in our network, it is desirable to increase the number of 1's in the input signal. In our architecture, neurons are activated by the presence of 1's, which

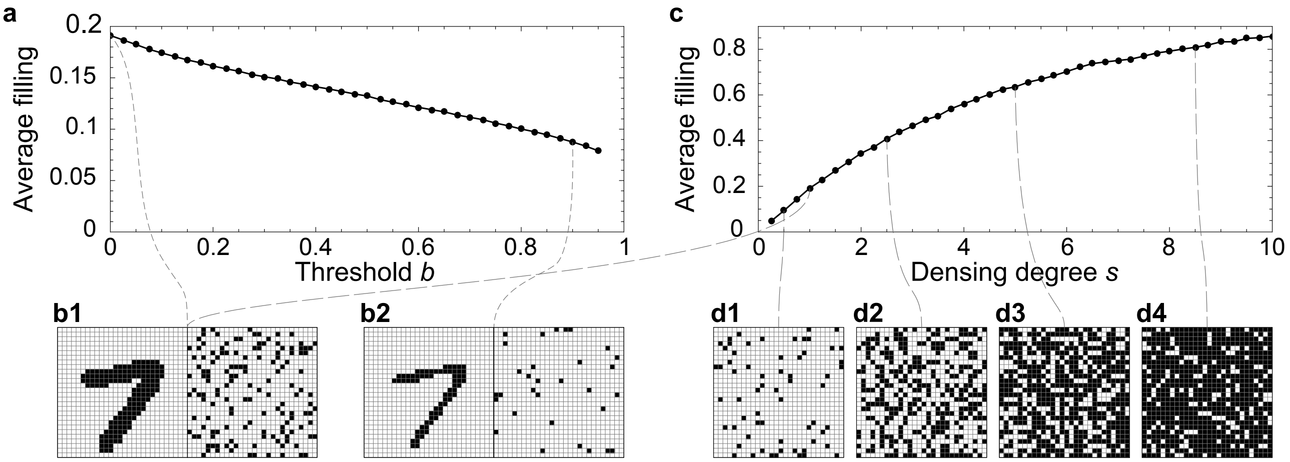

Figure S8: **Impact of threshold and densing degree on the filling of the input matrix.** **a** The dependence of the average filling of the input matrix on the threshold b at the densing degree $s = 1$ and **c** on the densing degree s at the threshold $b = 0$. **b**1,**b**2 Binarized input matrices before randomization (left panels) and after randomization (right panels) at the specified thresholds: $b = 0$ (**b**1) and $b = 0.9$ (**b**2). **d**1–**d**4 Binarized matrices after randomization at the specified densing degrees: $s = 0.5$ (**d**1), $s = 2.5$ (**d**2), $s = 5$ (**d**3) and $s = 8.5$ (**d**4)

facilitates their interactions and the manifestation of nonlinearity essential for complex computations. To achieve this increase in activation, we employ the input signal densing. This method involves overlapping multiple elements of the binarized image when mapping onto the input signal layer, so that each element in the input layer may represent a combination of several elements from the original image. As a result, the filling degree increases. See more details about the method in the main text. Figure S8**c** shows that the average filling degree increases monotonically with the densening degree s. Unlike threshold-based binarization, which is limited by the data's inherent structure, densing allows us to achieve any desired filling degree regardless of the original data. Examples of binarized images after randomization at different densing degrees are shown in Figs. S8**d**1–S8**d**4.

While it is technically possible to increase the filling degree to the maximum, doing so does not necessarily enhance the neural network's performance. In fact, extreme filling degrees, either 0 or 1, can lead to equally unrecognizable images. For instance, in Fig. S8**b**1, with a filling degree of approximately 0.2, the number of 1's is roughly equal to the number of 0's in Fig. S8**d**4 when the filling degree is about 0.8. If these images were to be classified directly by a linear classifier, they would be recognized with similar success, as such classifiers are insensitive to whether 0's or 1's dominate; a significant bias in either direction is functionally equivalent for them. However, in our network, the signal first passes through a layer of polariton neurons before reaching the linear classifier. This intermediate layer introduces asymmetry between filling degrees f and $(1 - f)$, affecting how the network processes the input data. Consequently, there exists an optimal filling degree that maximizes the network's performance by balancing neuron activation and ensuring effective nonlinear interactions.

S7 Strategies for scaling up polariton neural networks

To address the challenge of expanding the capacity of polariton neural networks beyond the current limitations, we propose several strategies. Each of these approaches aims to enhance the scalability of the system by overcoming spatial constraints and improving the efficiency of pump beam delivery.

S7.1 Tiling multiple spatial light modulators

Concept: The idea behind tiling multiple spatial light modulators (SLMs) is to increase the effective area that can be addressed by the pump beams, thereby enabling a larger network of neurons.

Details:

- *Modular Approach*: Instead of relying on a single SLM to cover the entire grid, multiple SLMs can be placed adjacent to each other to create a larger composite grid. This modular approach allows for the expansion of the addressable area beyond the physical limitations of a single SLM.
- *Alignment and Calibration*: Precise alignment of the tiled SLMs is crucial to ensure that the transition between adjacent SLMs is seamless. This can be achieved using high-precision positioning stages and alignment markers on the sample. Calibration procedures can help correct for any minor misalignments and ensure uniform beam intensity across the entire grid.
- *Synchronization*: The operation of multiple SLMs needs to be synchronized to maintain temporal coherence and uniformity of the pump pulses. Synchronization can be achieved through hardware controllers that coordinate the refresh rates and pulse timings of each SLM.

  To maintain the fast processing capabilities of the polariton network, optical synchronization techniques are employed. Ultrafast optical delay lines and beam splitters distribute a master synchronization pulse to all SLMs with femtosecond precision, eliminating latency associated with electronic components. Alternatively, optical phase-locked loops (OPLLs) can synchronize the phase of the laser beams driving each SLM, achieving attosecond precision. These methods ensure the required temporal coherence and uniformity across the tiled SLMs, preserving the rapid information processing speed of the polariton neural network without compromising its performance.
- *Optical Integration*: The optical setup must be designed to efficiently combine the output of multiple SLMs. This can be done using beam combiners, mirrors, and lenses to direct and focus the light from each SLM onto the appropriate regions of the sample. Careful design is required to minimize losses and ensure uniform illumination.

Advantages:

- *Scalability*: This approach allows the system to scale up to much larger networks by simply adding more SLMs to the array.
- *Flexibility*: Tiling SLMs provides flexibility in configuring the layout and density of the pump spots, allowing for tailored solutions based on specific experimental requirements.
- *Redundancy*: Using multiple SLMs can offer redundancy, where the failure of one SLM does not incapacitate the entire system, thus enhancing reliability.

Challenges:

- *Complexity*: The setup becomes more complex with the addition of multiple SLMs, requiring precise alignment and synchronization.
- *Cost*: Additional hardware components such as extra SLMs, alignment tools, and controllers increase the overall cost of the system.
- *Software Development*: Developing sophisticated control software to manage multiple SLMs and ensure seamless operation can be challenging and resource-intensive.

S7.2 Advanced Micro-Optics

Concept: Incorporating advanced micro-optics, such as microlens arrays, can help focus and direct the pump beams more precisely, allowing for denser packing of pump spots within the available area.

Details:

- *Microlens Arrays*: By using microlens arrays, one can achieve precise focusing and steering of the pump beams. These arrays consist of multiple tiny lenses that can be individually aligned to direct light to specific regions on the sample, allowing for tighter packing of pump spots.
- *Increased Packing Density*: The precise control provided by microlens arrays enables an increase in the density of the pump spots. This allows more neurons to be addressed within the same physical area, effectively expanding the capacity of the network without increasing the footprint.

- *Beam Shaping*: Advanced beam shaping techniques can be employed to optimize the distribution and intensity profile of the pump beams. Using diffractive optical elements (DOEs) in conjunction with microlens arrays, the beams can be shaped to match the specific requirements of the polariton condensates, enhancing their efficiency and effectiveness.
- *Optical Integration*: Integrating microlens arrays with other optical components, such as waveguides or beam splitters, can further improve the system's efficiency. This integration allows for more complex light routing and focusing schemes, enabling the precise delivery of light to the desired locations.
- *Fabrication Techniques*: Advanced fabrication techniques, such as photolithography and etching, enable the production of microlens arrays with high precision and uniformity. These techniques allow for the creation of custom lens arrays tailored to the specific requirements of the polariton neural network.

Advantages:

- *Higher Density*: Advanced micro-optics enable a higher density of pump spots, increasing the number of neurons that can be simultaneously addressed within a given area.
- *Precision*: The use of microlens arrays provides precise control over the beam placement and intensity, enhancing the overall performance of the neural network.
- *Scalability*: This approach supports the scalability of the system by allowing more neurons to be packed into the same physical space, facilitating the expansion of the network.
- *Customization*: Microlens arrays can be custom-designed to meet specific spatial and optical requirements, offering flexibility in the design and implementation of the neural network.

Challenges:

- *Complexity*: The design and fabrication of advanced micro-optics require high precision and sophisticated techniques, which can increase the complexity of the system.
- *Alignment*: Ensuring precise alignment of microlens arrays and other optical components is critical to maintaining performance and requires meticulous calibration.
- *Cost*: Advanced optical components and their integration can be expensive, potentially increasing the overall cost of the system.

S7.3 Optical Waveguides

Concept: Utilizing optical waveguides to deliver pump beams to specific locations on the sample can bypass the spatial limitations of free-space optics, enabling the creation of larger networks without increasing the physical footprint of the pump array.

Details:

- *Integration of Waveguides*: Optical waveguides can be integrated onto the sample to direct light precisely to designated spots. These waveguides can be fabricated using advanced lithography techniques to create paths for light with minimal loss and high precision.
- *Alignment and Calibration*: The alignment of waveguides must be precise to ensure that the light is delivered accurately to the intended locations. Calibration procedures can be used to correct any minor misalignments and to ensure uniform light intensity across the network. Waveguides can be designed to incorporate alignment markers and coupling structures to facilitate this process.
- *Waveguide Design*: The design of the waveguides must consider factors such as mode confinement, propagation loss, and bending radius. Optimizing these parameters ensures efficient light delivery and minimal signal degradation.
- *Coupling Efficiency*: Efficient coupling of light into and out of the waveguides is critical for maintaining the overall efficiency of the system. Techniques such as grating couplers or tapered structures can be used to enhance the coupling efficiency, ensuring that maximum light is delivered to the sample.

Advantages:

- *Scalability*: Optical waveguides enable the system to scale up to much larger networks by efficiently routing light to specific locations without increasing the physical footprint.
- *Precision*: Waveguides provide high precision in light delivery, ensuring accurate targeting of pump spots.
- *Efficiency*: Waveguides minimize light loss and ensure efficient delivery of pump pulses to the sample.

Challenges:

- *Fabrication Complexity*: The fabrication of high-performance waveguides requires advanced techniques and precise control over the fabrication process.
- *Integration*: Integrating waveguides with the existing optical setup and ensuring compatibility with other components can be challenging.

S7.4 Dynamic Reconfiguration

Concept: Implementing dynamic reconfiguration techniques allows a single SLM to sequentially address different regions of the sample at high speeds or to reproduce different configurations of the neural network on the same region. This time-multiplexing approach effectively increases the number of neurons that can be controlled without increasing the physical size of the SLM or the sample.

Details:

- *Time-Multiplexing Spatial Regions*: One strategy involves using time-multiplexing to rapidly switch the SLM between different spatial regions of the sample. The SLM addresses each region in sequence, allowing for the control of a much larger number of neurons than would be possible with static illumination. High-speed modulation and precise synchronization between the SLM and the pump pulses are essential to maintain coherence and uniformity.
- *Reproducing Neural Network Configurations*: Another strategy is to divide the neural network configuration into smaller fragments and sequentially project each fragment onto the same physical region of the sample. This approach reduces the required sample area and minimizes the amount of optical hardware needed, such as detectors. The SLM dynamically reconfigures to display different parts of the neural network project, addressing each fragment one after the other. This method requires precise timing and control to ensure the accurate reproduction of the neural network configurations.
- *High-Speed Modulation*: Both strategies require high-speed modulation of the SLM and the pump pulses to ensure that each region or configuration fragment is addressed within the appropriate time frame.
- *Synchronization*: Synchronization between the SLM and the pump laser is crucial. Both components must be precisely timed so that the SLM's pattern matches the timing of the pump pulses. Optical synchronization techniques, such as using a master clock signal, can ensure this precision without introducing significant delays.

Advantages:

- *Scalability*: Dynamic reconfiguration significantly increases the number of controllable neurons without requiring additional physical space for more SLMs.
- *Efficiency*: Efficient use of the SLM's capabilities maximizes the number of neurons that can be addressed, improving the overall system performance.
- *Flexibility*: This approach allows for flexible reconfiguration of the neuron layout and can adapt to different experimental requirements. The configuration reproduction strategy also reduces the physical size of the sample and the amount of required optical hardware.

Challenges:

- *Complexity*: Implementing dynamic reconfiguration adds complexity to the control system, requiring precise timing and coordination.
- *Speed Limitations*: The speed of the reconfiguration is limited by the SLM's refresh rate and the modulation capabilities of the pump laser.

S8 Energy Consumption and Efficiency Analysis of Polariton-Based Neuromorphic Networks

The energy efficiency of neuromorphic systems is often evaluated using the metric of Synaptic Operations per Second per Watt (SOPS). This metric provides a standardized way to compare different neuromorphic architectures in terms of their computational power relative to energy consumption. Our proposed polariton network leverages interactions between condensates and the resulting interference patterns, requiring energy input via pumping. To assess the minimal energy required effectively, it's essential to consider the theoretical minimal pump pulse energy necessary to induce noticeable interaction effects that could switch the parity of the interference pattern. Through theoretical estimates and corroborating literature, we deduce that the interaction between polaritons becomes substantial enough to influence system dynamics at a certain threshold of energy input. For example, the interaction effects between polaritons are significant when the potential barrier created by the exciton reservoir is sufficiently robust to alter the coherence properties of the polariton condensates. By employing the nonlinear coefficient for polaritons, approximately $2.4\,\mu\text{eV}\cdot\mu\text{m}^2$, and a lowest polariton density achievable of $10^8\,\text{cm}^{-2}$ [S5], we calculate the minimal energy needed for these effects. If we consider that only about 10% of the non-resonant pump energy is effectively converted into polariton energy due to various inefficiencies (thermal losses, scattering, etc.), the minimal pump pulse energy of about 0.4 fJ per condensate should suffice. For a dyad consisting of two such condensates, this would total about 0.8 fJ.

Calculating the power consumption for these pulses, given a pulse duration (single synaptic operation) of $\tau_1 = 5$ ps, the power used per pump pulse amounts to: $P_{\text{pump}} = 0.16\,\text{mW}$. In the proposed network architecture, each neuron is indirectly connected to a number of other neurons, ranging from 0 to 2. For estimations, it is assumed that on average, each neuron is connected to one other neuron. Under this assumption, the total number of synaptic connections in the network, denoted as N_{ns}, can be equated to the total number of neurons, $N_{\text{ns}} = N_{\text{d}}$. Thus, the SOPS for generating one neuron, without considering input signals, is

$$\text{SOPS}_{\text{pump}} = \frac{N_{\text{ns}}}{\tau_1 N_{\text{d}} P_{\text{pump}}} = 1.25 \times 10^{15}\,\text{s}^{-1}\text{W}^{-1}.$$

Considering the requirements for switching neurons between OFF and ON states, which also necessitate signal pulses, the dynamics change slightly. Assuming the signal pulse energy is about one-third that of the pump pulse and considering a pulse duration of $\tau_2 = 8$ ps, the additional power required can be approximated as $P_{\text{signal}} = 0.027\,\text{mW}$. Total power consumption, including both pump and signal contributions is then estimated as about $P_{\text{total}} = 0.19\,\text{mW}$. The overall SOPS, including both pump and signal contributions, recalculates to

$$\text{SOPS}_{\text{total}} = \frac{N_{\text{ns}}}{\tau_2 N_{\text{d}} P_{\text{total}}} \approx 6.6 \times 10^{14}\,\text{s}^{-1}\ \text{W}^{-1}.$$

The signal densing technique proposed in the manuscript contributes to the reduction of the network's energy consumption by enhancing computational efficiency. Without signal densing, a maximum classification accuracy of 95% is achieved with approximately 10^4 neurons. However, with a signal densing degree of around 3, this accuracy can be reached with about 800 neurons—more than an order of magnitude fewer, leading to a proportional reduction in energy consumption.

In their study [S6], the authors propose an even lower polariton density threshold required to exhibit nonlinearity, potentially allowing further reduction in the energy per pulse. They also suggest a resonant pumping regime that could enhance the efficiency of energy transfer to the condensate. According to their estimates, these adjustments could lower the required pulse energy by three orders of magnitude, correspondingly increasing the SOPS significantly. While the potential to enhance our system's efficiency is impressive, we will adhere to more conservative estimates in our analysis.

The use of active optical elements such as SLMs is not essential in our system. This functionality can be replaced by passive optical elements that do not require energy to operate, such as arrays of microlenses, optical waveguides, and static diffractive optical elements. Similarly, the effective potential for the localization and isolation of dyads can be formed using methods like deep etching techniques and dissipation boundaries, which do not require continuous energy input. However, for proof-of-principle demonstrations and optimization of the lattice geometry in laboratory experiments, SLMs indeed can be used. The energy consumption of an SLM setup, operating at typical efficiency levels, is in the range of 5 mJ per second for a full 28x28 pixel grid at a switching frequency of 1 kHz.

Regarding the energy necessary for the conversion between electronic and optical signals, this conversion typically involves devices such as electro-optic modulators or photodetectors. For electro-optic modulators, the

System	SOPS (s^{-1} W^{-1})	Energy Consumption	Processing Speed
Our system	6.6×10^{14}	0.4 fJ*	tens of picoseconds**
TrueNorth [S7]	4.5×10^{10}	26 pJ	1 ms
SpiNNaker [S8]	1.3×10^{8}	8 nJ	26 ms
HiAER-IFAT [S9, S10]	4.5×10^{10}	22 pJ	14 ns
HI-CANN [S11]	1.4×10^{8}	7.4 nJ	5 ns
Neurogrid [S12]	10^{9}	0.94 nJ	0.1 ns
Superconducting Nanowires [S13]	5×10^{14}	5×10^{-2} fJ	1 ns
FireFly [S14]	2×10^{12}	0.5 pJ	3 ns
Silicon photonic circuit [S15]	$2 \times 5 \times 10^{13}$	20 fJ	1 ns

Table S1: Comparative analysis of neuromorphic systems

*Does not account for performance and inefficiencies of the pumping system.

**The processing speed is determined by the lifetime of polaritons, excited by a single-shot laser pulse, and does not account for possible delays caused by electronic components.

energy per bit is generally around 1 pJ, while for photodetectors, it is slightly lower, around 0.5 pJ per bit. For the energy implicated in memory access, modern memory technologies, such as DRAM, consume approximately 1–10 nJ per access. This value varies depending on the specific technology and configuration but provides a reasonable estimate for typical memory operations.

Based on the estimations above, we can estimate the lower and upper boundaries of energy consumption for our system. In an ideal scenario utilizing only passive optical elements and efficient energy use in the conversion and memory access stages, the energy consumption can be minimized to approximately 0.1 nJ per synaptic operation. This includes contributions from both the condensate pumping and the signal conversion stages. When including the additional overhead from using SLMs and less efficient components, the energy consumption could rise to about 1 nJ per synaptic operation. This estimate accounts for higher energy usage in the SLM operation and less optimized conversion processes.

Table S1 provides a comparative analysis of various hardware implementations of neuromorphic systems based on key metrics: SOPS, energy consumption per operation, and processing speed. Our proposed system could potentially demonstrates a considerable advancement with all the characteristics. This table underscores the superior energy efficiency and processing capabilities of our system relative to existing technologies. More systematic and detailed information on various neuromorphic systems can be found in the comprehensive review [S16].

S9 Schematic Overview of Proposed Experimental Implementation for Polariton-Based Neuromorphic Networks

In this section, we provide a schematic and description of the experimental setup designed to demonstrate the feasibility of our proposed polariton-based neuromorphic network, see Fig. S9. The experimental configuration begins with a laser operating in pulse regime, emitting coherent pump pulses essential for initiating polariton states within the microcavity sample. These laser pulses (depicted in red) in the schematic, are directed towards a light modulation device. The modulation of light adjusts the intensity of the incoming laser pulses to create both the input signals and the neuronal condensate states. As discussed earlier, this modulation can be implemented using various techniques. For demonstration purposes, SLM can be employed due to its versatility and precision in controlling light characteristics. Alternatively, the setup could incorporate arrays of microlenses or waveguide arrays, capable of manipulating light paths and intensities through fixed, passive optical paths.

In this configuration, the system inherently operates with laser pulses of the same duration for both signal generation and polariton condensate formation within the dyads. Our calculations confirm that using a single pulse duration is feasible for implementing the desired functionalities of our neuromorphic network. However, to enable different temporal characteristics for pulses intended for varied tasks, the experimental setup could be augmented. This enhancement might include an additional laser source or a modulating device at the open input of the first beam splitter.

After modulation, the pump radiation (now depicted in blue) travels to the microcavity sample. This

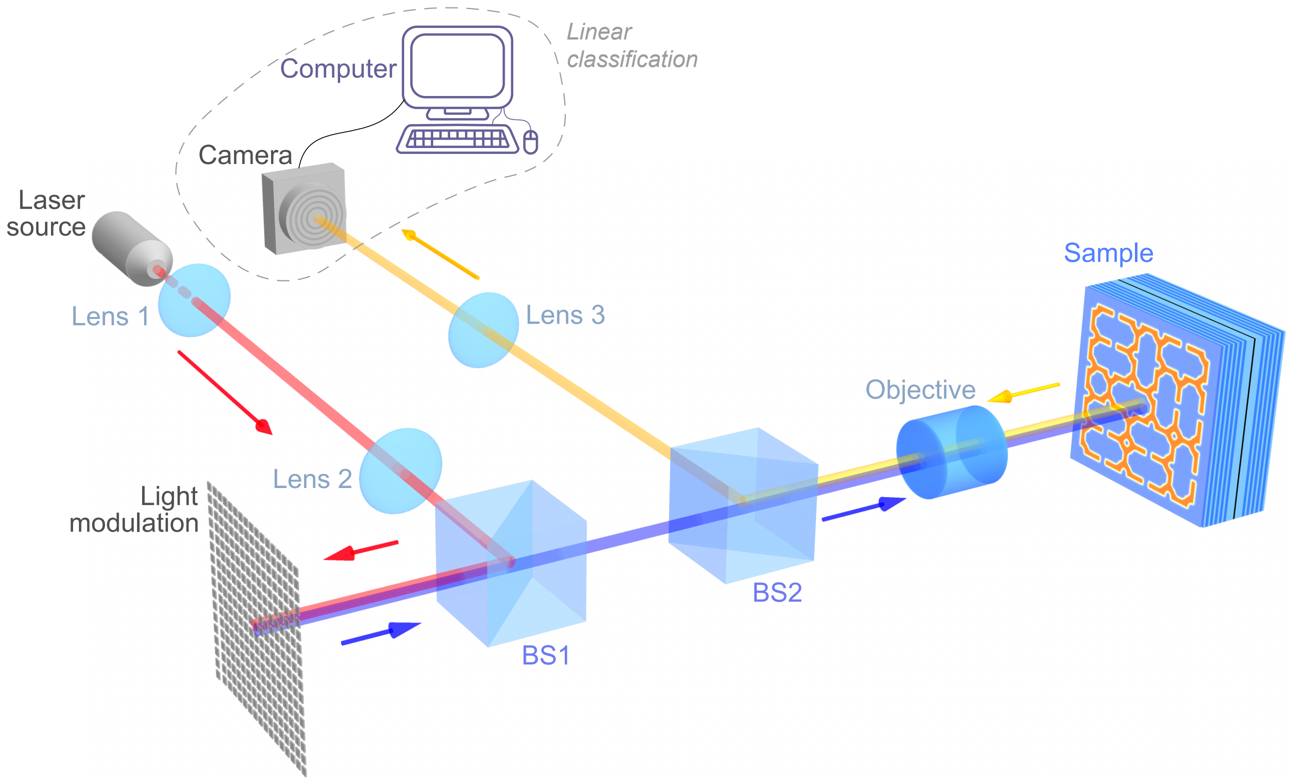

Figure S9: **Schematic diagram of the possible experimental setup for a polariton-based neuromorphic network.** A laser source emits a pulse (red line) that is directed through a beam splitter, to a light modulation device where it is modulated to encode the lattice of polariton dyads and the input data. The modulated light (blue line) interacts with the microcavity sample, generating polariton condensates. Resulting photoluminescence (orange line) is captured by a camera, the output of which is analyzed by a connected computer acting as a linear classifier

interaction is crucial as it activates the polariton condensates, which are arranged in a predefined lattice structure within the sample. The arrangement and interaction of these condensates under light modulation perform the computational processes.

The optical pathway in the setup includes three lenses and an objective. A pair of lenses, L1 and L2, arranged in a $4f$ configuration is placed after the light source to ensure that the light is evenly spread over the light modulation device. An objective added between the beam splitter BS2 and the sample serves both to focus the modulated beam onto the sample and to collect the emitted photoluminescence from the sample. Lens L3, used in conjunction with the objective, captures the far-field pattern of the light to provide a real space image of the polariton modes.

The photoluminescence emitted by the polaritons (depicted in orange) represents the output data. In the described setup, the combination of a camera and a computer is primarily utilized to demonstrate the proof of principle of the polariton neural network. The camera captures the photoluminescence pattern, and the computer processes these images as a linear classifier, translating the optical patterns into computational outcomes based on the network's response to the input signals. However, it's important to note that the linear classification can be implemented entirely optically. This can be achieved through the use of an optical correlator that integrates diffractive optical elements to perform Fourier transformations on the incoming light patterns. By aligning specific phase and amplitude patterns corresponding to the expected photoluminescence signals, this optical system can directly correlate input patterns to stored reference patterns, thereby accomplishing classification without the need for electronic processing.

References

[S1] Davis, S. and Mermelstein, P., IEEE Transactions on Acoustics, Speech, and Signal Processing **28**, 357 (1980).

[S2] Md. Sahidullah and Goutam Saha, Speech Communication **54**, 543 (2012).

[S3] Pete Warden, "Speech commands: A dataset for limited-vocabulary speech recognition," (2018), arXiv:1804.03209 [cs.CL] .

[S4] Speech commands dataset version 2. [Online]. Available: http://download.tensorflow.org/data/speech_commands_v0.02.tar.gz. Released under the Creative Commons BY 4.0 license [Online]. Available: https://creativecommons.org/licenses/by/4.0/.

[S5] Y. Luo, Q. Guo, X. Deng, S. Ghosh, Q. Zhang, H. Xu, and Q. Xiong, Light: Science & Applications **12**, 220 (2023).

[S6] M. Matuszewski, A. Opala, R. Mirek, M. Furman, M. Król, K. Tyszka, T. Liew, D. Ballarini, D. Sanvitto, J. Szczytko, and B. Pietka, Phys. Rev. Appl. **16**, 024045 (2021).

[S7] P. A. Merolla, J. V. Arthur, R. Alvarez-Icaza, A. S. Cassidy, J. Sawada, F. Akopyan, B. L. Jackson, N. Imam, C. Guo, Y. Nakamura, B. Brezzo, I. Vo, S. K. Esser, R. Appuswamy, B. Taba, A. Amir, M. D. Flickner, W. P. Risk, R. Manohar, and D. S. Modha, Science **345**, 668 (2014).

[S8] E. Stromatias, F. Galluppi, C. Patterson, and S. Furber, in *The 2013 International Joint Conference on Neural Networks (IJCNN)* (2013) pp. 1–8.

[S9] T. Yu, J. Park, S. Joshi, C. Maier, and G. Cauwenberghs, in *2012 IEEE Biomedical Circuits and Systems Conference (BioCAS)* (2012) pp. 21–24.

[S10] J. Park, S. Ha, T. Yu, E. Neftci, and G. Cauwenberghs, in *2014 IEEE Biomedical Circuits and Systems Conference (BioCAS) Proceedings* (2014) pp. 675–678.

[S11] J. Schemmel, D. Brüderle, A. Grübl, M. Hock, K. Meier, and S. Millner, in *2010 IEEE International Symposium on Circuits and Systems (ISCAS)* (2010) pp. 1947–1950.

[S12] B. V. Benjamin, P. Gao, E. McQuinn, S. Choudhary, A. R. Chandrasekaran, J.-M. Bussat, R. Alvarez-Icaza, J. V. Arthur, P. A. Merolla, and K. Boahen, Proceedings of the IEEE **102**, 699 (2014).

[S13] E. Toomey, K. Segall, and K. K. Berggren, Frontiers in Neuroscience **13** (2019), 10.3389/fnins.2019.00933.

[S14] J. Li, G. Shen, D. Zhao, Q. Zhang, and Y. Zeng, IEEE Transactions on Very Large Scale Integration (VLSI) Systems **31**, 1178 (2023).

[S15] A. N. Tait, T. F. de Lima, E. Zhou, A. X. Wu, M. A. Nahmias, B. J. Shastri, and P. R. Prucnal, Scientific Reports **7**, 7430 (2017).

[S16] J. Zhu, T. Zhang, Y. Yang, and R. Huang, Applied Physics Reviews **7**, 011312 (2020).
